# Renal response to short- and long-term exercise in very-long-chain acyl-CoA dehydrogenase-deficient (VLCAD^−/−^) mice

**DOI:** 10.1186/s40348-014-0005-z

**Published:** 2014-10-02

**Authors:** Sara Tucci, Antonia Krogmann, Diran Herebian, Ute Spiekerkoetter

**Affiliations:** Department of General Pediatrics, Center for Pediatrics and Adolescent Medicine, University Hospital Freiburg, Mathildenstrasse 1, Freiburg, 79106 Germany; Department of General Pediatrics, Neonatology and Children′s Cardiology, University Children′s Hospital, Duesseldorf, 40225 Germany

**Keywords:** VLCAD deficiency, Fatty acid oxidation, Acylcarnitines, Glucose homeostasis, Oxidative stress

## Abstract

**Background:**

Deficiency of very long-chain acyl-CoA dehydrogenase (VLCAD) is the most common disorder of mitochondrial β-oxidation of long-chain fatty acids. In order to maintain glucose homeostasis, the kidney and liver as the main gluconeogenic organs play an important role under conditions of impaired fatty acid oxidation. However, little is known about how a defective fatty acid oxidation machinery affects renal metabolism and function as well as renal energy supply especially during catabolic situations.

**Methods:**

In this study, we analyzed VLCAD^−/−^ mice under different metabolic conditions such as after moderate (1 h) and intensive long-term (1 h twice per day over 2 weeks) physical exercise and after 24 h of fasting. We measured the oxidation rate of palmitoyl-CoA (C16-CoA) as well as the expression of genes involved in lipogenesis and renal failure. Oxidative stress was assessed by the function of antioxidant enzymes. Moreover, we quantified the content of glycogen and long-chain acylcarnitines in the kidney.

**Results:**

We observed a significant depletion in renal glycogen with a concomitant reduction in long-chain acylcarnitines, suggesting a substrate switch for energy production and an optimal compensation of impaired fatty acid oxidation in the kidney. In fact, the mutants did not show any signs of oxidative stress or renal failure under catabolic conditions.

**Conclusions:**

Our data demonstrate that despite *Acadvl* ablation, the kidney of VLCAD^−/−^ mice fully compensates for impaired fatty acid oxidation by enhanced glycogen utilization and preserves renal energy metabolism and function.

## Background

Deficiency of the very long-chain acyl-CoA dehydrogenase (VLCAD) is the most common disorder of mitochondrial β-oxidation of long-chain fatty acids with an incidence of 1:30,000 to 1:100,000 newborns [1,2]. The clinical phenotype of VLCAD deficiency (VLCADD) is very heterogeneous and presents with different severity and age of onset [3]. The symptoms usually manifest in situations of increased energy demand such as fasting, infectious illnesses, and intensive or prolonged physical exercise when the organism relies on fatty acid β-oxidation for energy supply. Before the implementation of newborn screening programs for fatty acid oxidation defects, three different phenotypes could be identified. The most severe phenotype appeared in the first weeks and months of life and presented with hypoketotic hypoglycemia, hypertrophic cardiomyopathy, and encephalopathy. The infantile hepatic phenotype was triggered by infections and presented with hypoketotic hypoglycemia, hepatopathy, and lethargy. The milder later-onset myopathic phenotype was characterized by muscle weakness, rhabdomyolysis, and myoglobinuria in adolescents or young adults [4,5].

The VLCAD^−/−^ mouse is a reliable model and develops symptoms during catabolic situations as occur in humans with a milder later-onset phenotype [6-12]. Indeed, although asymptomatic under resting conditions, VLCAD^−/−^ mice present with hypoglycemia, hepatopathy, and skeletal myopathy during catabolism [9,13]. Compensatory mechanisms based on enhanced glucose oxidation are effective and supply sufficient energy at rest [14-16]. The role of the kidneys in providing energy as a gluconeogenic organ such as the liver has not been studied yet. Therefore, we here investigated how a defective fatty acid oxidation machinery affects kidney function and kidney metabolism with special focus on glucose supply. To address this question, we measured the palmitoyl-CoA (C16-CoA) oxidation rate in the kidney of wild-type (WT) and VLCAD^−/−^ mice in situations of normal and increased energy demand as well as the expression of other dehydrogenases with overlapping substrate specificity. Renal damage and function were characterized by the activity of antioxidant enzymes and by the expression of genes upregulated immediately prior to renal failure. Finally, renal glycogen content and acylcarnitines as markers for impaired energy production from fatty acid oxidation were quantified.

## Methods

### Animals

VLCAD^−/−^ mice have been generated as described in detail by Exil et al. [13]. Experiments were performed on fourth- to fifth-generation intercrosses of C57BL6+129sv VLCAD genotypes. Littermates served as controls, and genotyping of mice was performed as described previously [13]. Groups consisting of 5 to 6 mice of both genders, at the age of 12 to 13 weeks, were investigated under resting and different catabolic conditions. The mice were sacrificed at the end of the experiment by CO_2_ asphyxiation. The kidneys were rapidly removed and immediately frozen in liquid nitrogen. The experiments have been performed twice. All animal studies were performed with the approval of the University Institutional Animal Care and Use Committee and in accordance with the Committees' (LANUV) guidelines.

### Diet composition, exercise protocols, and fasting

After weaning, the mice of each genotype were fed a normal purified mouse diet containing 5% crude fat in form of LCT, corresponding to 12% of metabolizable energy as calculated with Atwater factors (ssniff® EF R/M Control, ssniff Spezialdiäten GmbH, Soest, Germany). All mice groups received water *ad libitum*. The mice were analyzed under well-fed conditions or after 24 h of fasting.

As mice are nocturnal animals, treadmill running was performed during the dark cycle. To analyze the effects of different exercise bouts, one group of WT and VLCAD^−/−^ mice was exercised for 60 min once. The mice were placed in an exercise chamber, and after an adaptation period of 15 min, initial belt speed was set to 4 m/min and increased every 5 min by 2 m/min to a maximum of 16 m/min. The mice were exercised until they displayed signs of exhaustion (>2 s spent on the shocker plate without attempting to re-engage the treadmill) or the exercise was terminated after 60 min. The second group of mice was subjected to a long-term exercise stress. The animals had to run 45 min twice a day for 2 weeks (5 days per week) at 0° inclination. The long-term exercise protocol started with a running speed of 10 m/min. At day 5, the running speed was reduced to 8 m/min until day 10 and further reduced to 6 m/min at day 11 until the end of the experiment. Both experiments were conducted on a Columbus Instruments Simplex II metabolic rodent treadmill (Columbus, OH, USA) consisting of four individual lanes without inclination and a shock plate incentive (3 Hz, 200 ms, 160 V, 1.5 mA).

### Tissue homogenates and protein expression

The tissues were homogenized in Cellytic MT Buffer (Sigma-Aldrich, Steinheim, Germany) in the presence of 1 mg/ml protease inhibitors and centrifuged at 4°C and 16,000 × *g* for 10 min to pelletize any cell debris. The clear supernatant was immediately used for the enzyme assays or stored at −80°C. The protein concentration of tissue homogenates was determined using the BSA method as described previously [17].

### Oxidation rate and analysis of palmitoyl-CoA by LC-MS/MS

The oxidation rate of C16-CoA was measured as reported previously [15]. C16-CoA, was purchased from Sigma (Steinheim, Germany). Reaction products were detected by LC-ESI-MS/MS in positive ionization mode according to the published literature [15].

### Enzyme activities

Catalase, glutathione peroxidase (GPX), and NAD(P)H: quinone oxidoreductase were measured to determine the development and cellular localization of oxidative stress. The peroxisomal catalase activity was measured fluorometrically by the production of the highly fluorescent oxidation product resorufin [18]. The mitochondrial glutathione peroxidase activity was determined by calculating the oxidation rate of nicotinamide adenine dinucleotide phosphate (NADPH) to NADP^+^ spectrophotometrically at 340 nm for 4 min as previously described [19,20]. The NAD(P)H: quinone oxidoreductase, marker for cytosolic oxidative stress was measured as described by Milder et al. [21]. Briefly, the reactions contained 25 mM Tris-HCl (pH 7.4), 0.7 mg/ml bovine serum albumin, and 0.2 mM NADH. The reactions were started by the addition of 40 μM 2,6-dichlorophenol-indophenol. The reactions were performed in the absence and presence of 20 μM dicumarol. NQO1 activity is defined as the dicumarol-inhibitable reduction of 2,6-dichlorophenolindophenol measured at 600 nm at 30°C.

### Analysis of acylcarnitines

The analysis of long-chain acylcarnitines was performed in the kidney as previously described [22,23]. Briefly, the acylcarnitines were extracted from the tissues with acetonitrile/water (80%/20% *v*/*v*) in the presence of [^2^H_3_] free carnitine, [^2^H_3_] octanoyl-carnitine, and [^2^H_3_] palmitoyl-carnitine as internal standard. The extracted supernatant was dried, and the butylated acylcarnitines were analyzed by electron spray ionization tandem mass spectrometry (ESI-MS/MS). All even-chain C14-C18 acylcarnitines (saturated and unsaturated) were measured.

### Extraction and analysis of renal glycogen

The glycogen concentrations of the kidney were quantified as duplicates by using an enzymatic kit (EnzyChrom™ Glycogen Assay Kit, BioTrend, Cologne, Germany) on an Infinite M200 Tecan (Crailsheim, Germany) plate reader. The assays were performed following the manufacturer's instructions.

### Real-time PCR analysis

Total renal RNA was isolated with the RNeasy mini kit (Qiagen, Hilden, Germany). Forward and reverse primers for β-actin, acyl-CoA oxidase (*AOX*), lipocalin 2 (*Ngal*), kidney injury molecule 1 (*KIM1*), heme oxygenase 1 (*Ho1*), stearoyl element binding protein-1c (*SREBP-1c*), fatty acid synthase (*FASN*), and acetyl-CoA carboxylase 1α (*ACC1α*) were designed with the FastPCR program (R. Kalendar, Institute of Biotechnology, Helsinki, Finland). Gene function and primer sequences are reported in Table 1. Real-time PCR was performed in a single-step procedure with the QuantiTect SYBR Green™ RT-PCR (Qiagen, Hilden, Germany) on an Applied Biosystems 7500 Sequence Detection System in Micro Amp 96-well optical reaction plates capped with MicroAmp optical caps (Applied Biosystems, Foster City, CA, USA). The values in all samples were normalized to the expression level of β-actin as internal standard.

**Table 1:** **Gene function and primer sequences**

**Probe**	**Forward 5′ → 3′**	**Reverse 5′ → 3′**	**GenBank**
β-actin	TAGGCACCAGGGTGTGATGG	CTCCATGTCGTCCCAGTTGG	NM_009606.2
β-oxidation			
*MCAD*	GAAAGTTGCGGTGGCCTTGG	AAGCACACATCATTGGCTGC	NM_007382.4
*LCAD*	GGGAAGAGCAAGCGTACTCC	TCTGTCATGGCTATGGCACC	NM_007381.3
*AOX*	TGCCCAGGTGAGAAGCCTGACG	TCAGACTGGCGCCTCACAGC	NM_015729.2
Renal failure			
*Ngal*	GTGGTACGTTGTGGGCCTGG	TGGCCAGCCCTGGAGCTTGG	NM_008491
*KIM1*	GTCAGCATCTCTAAGCGTGG	GGCAGAAGGTCCCTCAGAGG	NM_001166631
*Ho1*	ATTGAGCTGTTTGAGGAGCTGC	CACTGCCACTGTTGCCAACAGG	NM_010442.2
Lipogenesis			
*SREBP-1c*	CAGCTCAGAGCCGTGGTGA	TTGATAGAAGACCGGTAGCGC	NM_01148
*ACC1α*	TCAACAGGCTGAGCTTCACACG	ACTGGTCATGATATCCTGCAGC	NM_133360
*FASN*	TCTGGAATCCGCACCGGCTACC	TTCCCGGGTTGCCCTGTCAAGG	NM_007988.3

### Statistical analysis

All data are presented as mean ± standard error of the mean (SEM). All data were tested with the Kolmogorov-Smirnov test and the Levene's test to investigate the Gaussian distribution and the homoscedasticity. Statistical analysis of differences between two means was assessed by unpaired Student's *t* test to correct for multiple comparisons using the Holm-Sidak method. Multiple means were compared by a two-way analysis of the variance (ANOVA) to test the effects of genotype and applied stress (GraphPad Prism 6.0, San Diego, CA, USA). A probability level of *p* < 0.05 was regarded as significant.

## Results

### Turnover rate of palmitoyl-CoA oxidation and gene expression of different dehydrogenases

**Figure 1:**
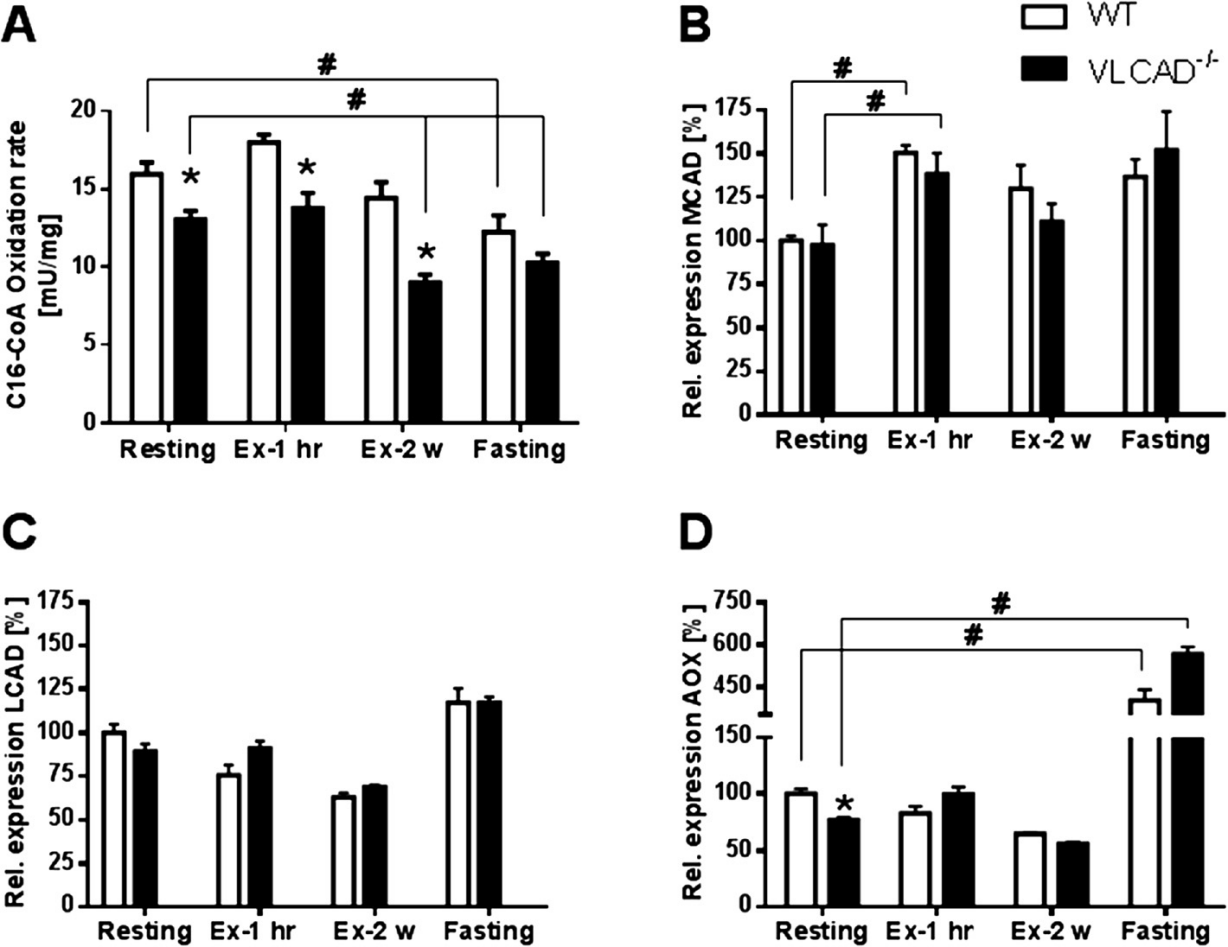
**Oxidation rate of palmitoyl-CoA (C16-CoA) (A) and gene expression of fatty acid dehydrogenases [MCAD (B), LCAD (C) and AOX (D)].** White and black bars represent WT and VLCAD-/- mice, respectively. Values are represented as mean ± SEM (n = 5-6).* indicates significant differences between WT and VLCAD-/- mice within an experimental set. # indicates significant differences between WT or VLCAD-/- mice under different stress conditions as compared to resting mice. * and # values were considered significant if p < 0.05 (Two way ANOVA with Bonferroni correction and Student’s t-test).

To test whether the oxidation rate in the kidney of VLCAD^−/−^ mice is influenced by increased energy demand or fasting, we measured the turnover rate of C16-CoA in mice during different conditions. As shown in Figure 1A, under resting condition, the turnover rate in VLCAD^−/−^ mice was significantly reduced by 20% in mutants as compared to the littermates. Interestingly, 1 h on the treadmill did not affect the oxidation capacity of the VLCAD^−/−^ mice, whereas a training protocol over 2 weeks significantly reduced the turnover rate of C16-CoA in the VLCAD^−/−^ mice as compared to sedentary mutants (9 ± 0.49 vs. 13.1 ± 0.54 mU/mg). A similar significant reduction of C16-CoA oxidation capacity was also observed after 24 h of fasting in both genotypes, as shown in Figure 1. Fasting, therefore, did not result in a genotype-specific effect in contrast to long-term physical exercise.

We also did not observe a genotype-specific effect on the gene expression of other dehydrogenases with partly overlapping substrate specificity to VLCAD, the mitochondrial *MCAD* and *LCAD* as well as the peroxisomal *AOX*. However, we observed a remarkable upregulation of *AOX* in both genotypes as adaptive response to fasting [24] (Figure 1D). Of particular interest was the significant reduction of C16-CoA oxidation rate in VLCAD^−/−^ mice after long-term physical exercise as compared to mutants under resting condition, suggesting enhanced energy production from glucose.

### Lipid accumulation and oxidative stress

**Figure 2:**
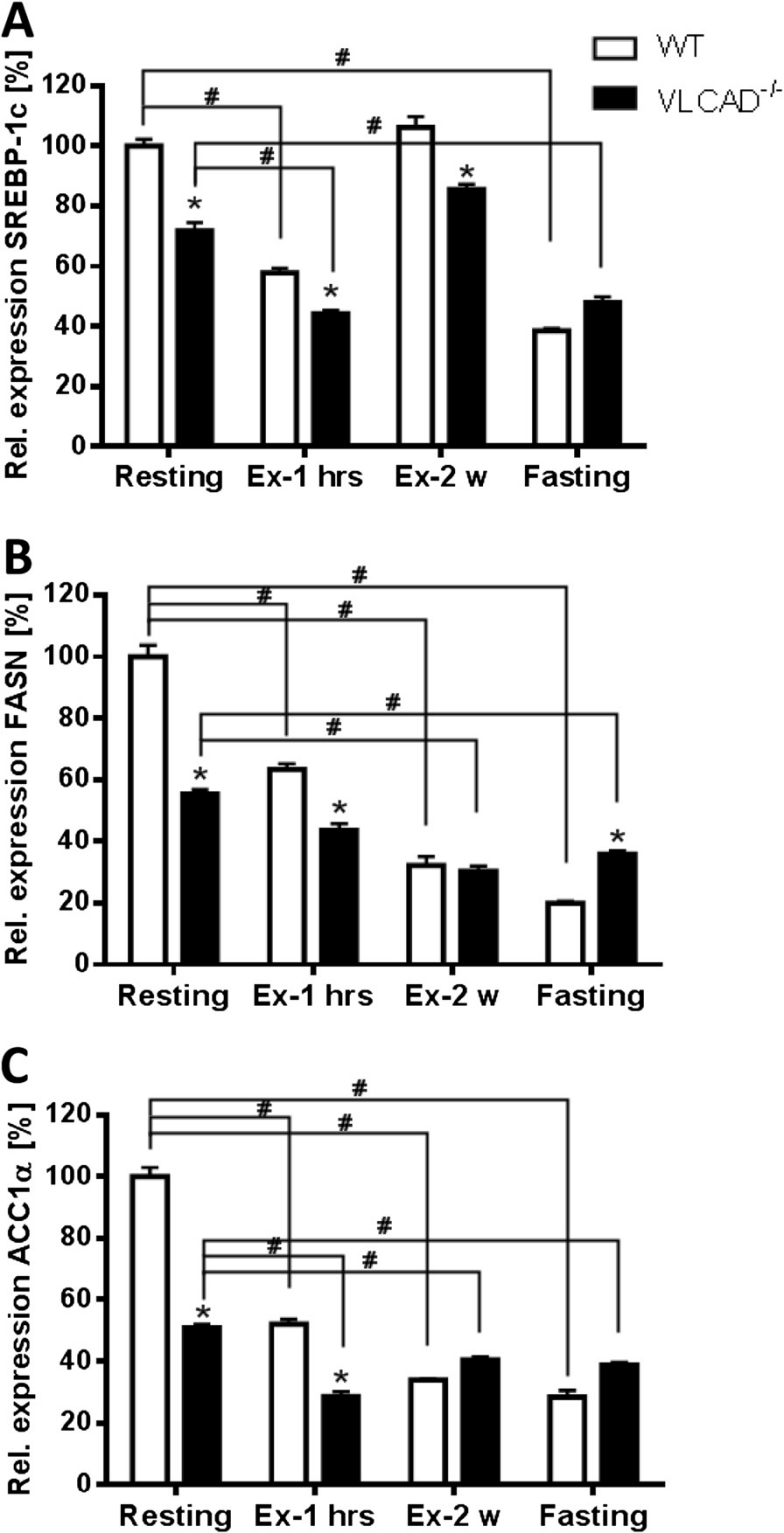
**Renal lipogenesis is not affected under different stress conditions. (A)** *SREBP-1c*, stearoyl-regulatory element binding protein 1c. **(B)** *FASN*, fatty acid synthetase. **(C)** *ACC1α*, acetyl-CoA carboxylase α. White and black bars represent WT and VLCAD^−/−^ mice, respectively. Values are represented as mean ± SEM (*n* = 5 to 6). Asterisk indicates significant differences between WT and VLCAD^−/−^ mice within an experimental set. Number sign indicates significant differences between WT or VLCAD^−/−^ mice under different stress conditions as compared to resting mice. Asterisk and number sign denote that values were considered significant if *p* < 0.05 (two-way ANOVA with Bonferroni correction and Student's *t* test).

Because catabolism stimulates lipolysis resulting in marked lipid accumulation in different organs of VLCAD^−/−^ mice [11], we measured the renal lipogenesis in all groups. We therefore tested the expression at mRNA level of genes involved in this pathway, such as the transcription factor *SREBP-1c*, which regulates lipid homeostasis, fatty acid biosynthesis, and glucose metabolism [25], as well as of its target genes *ACC1α* and *FASN*, responsible for *de novo* biosynthesis and elongation of fatty acids as they directly correlate with the triglyceride accumulation in the kidney [26]. As shown in Figure 2A,B,C, in contrast to results previously obtained from the liver [11,12], the expression of these genes was strongly downregulated in all groups independently of the applied stress.

**Figure 3:**
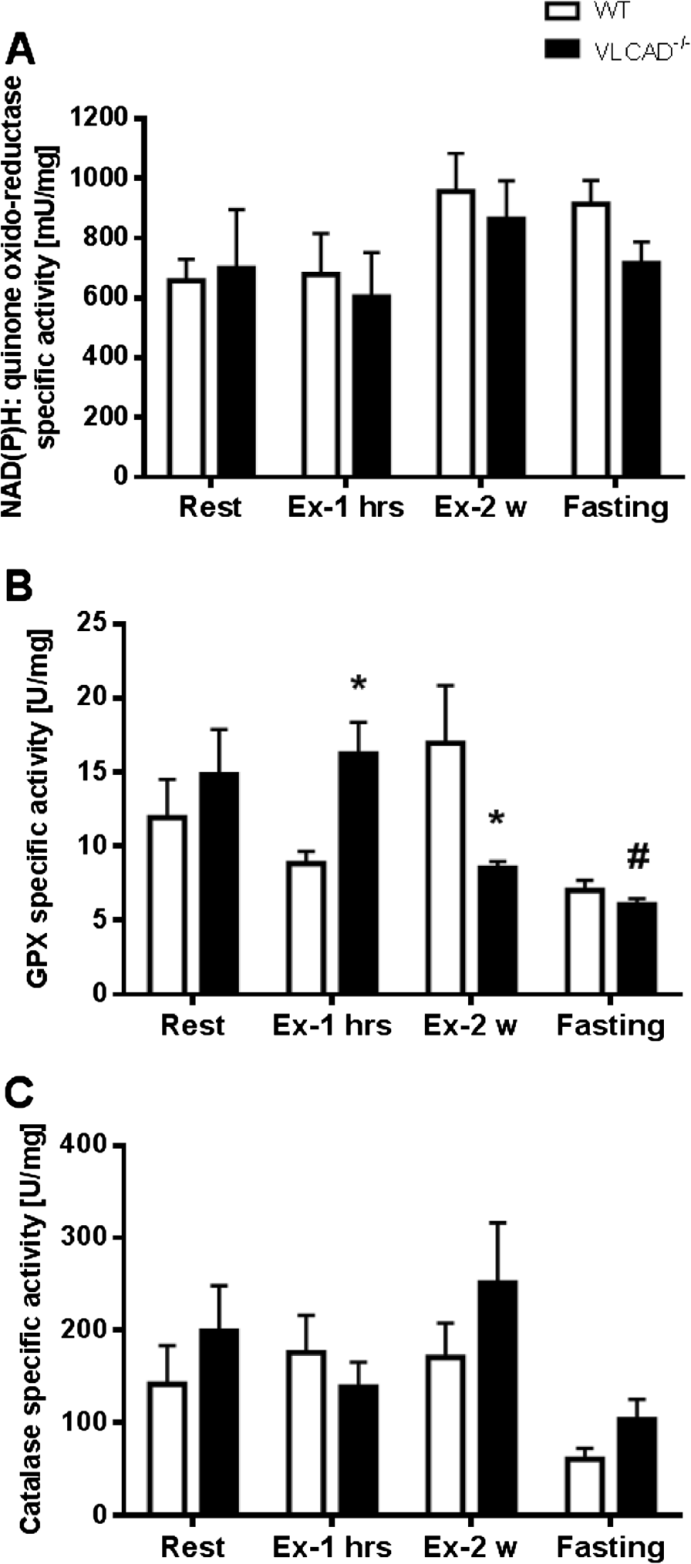
**Renal markers for oxidative stress in WT and VLCAD** ^**−/−**^ **mice under different stress conditions.** White and black bars represent WT and VLCAD^−/−^ mice, respectively. Values are represented as mean ± SEM (*n* = 5 to 6). Asterisk indicates significant differences between WT and VLCAD^−/−^ mice within an experimental set. Number sign indicates significant differences between WT or VLCAD^−/−^ mice under different stress conditions as compared to resting mice. Asterisk and number sign denote that values were considered significant if *p* < 0.05 (two-way ANOVA with Bonferroni correction and Student's *t* test). Specific activities of **(A)** NAD(P)H: quinone oxidoreductase, **(B)** GPX, and **(C)** catalase under different stress conditions.

**Figure 4:**
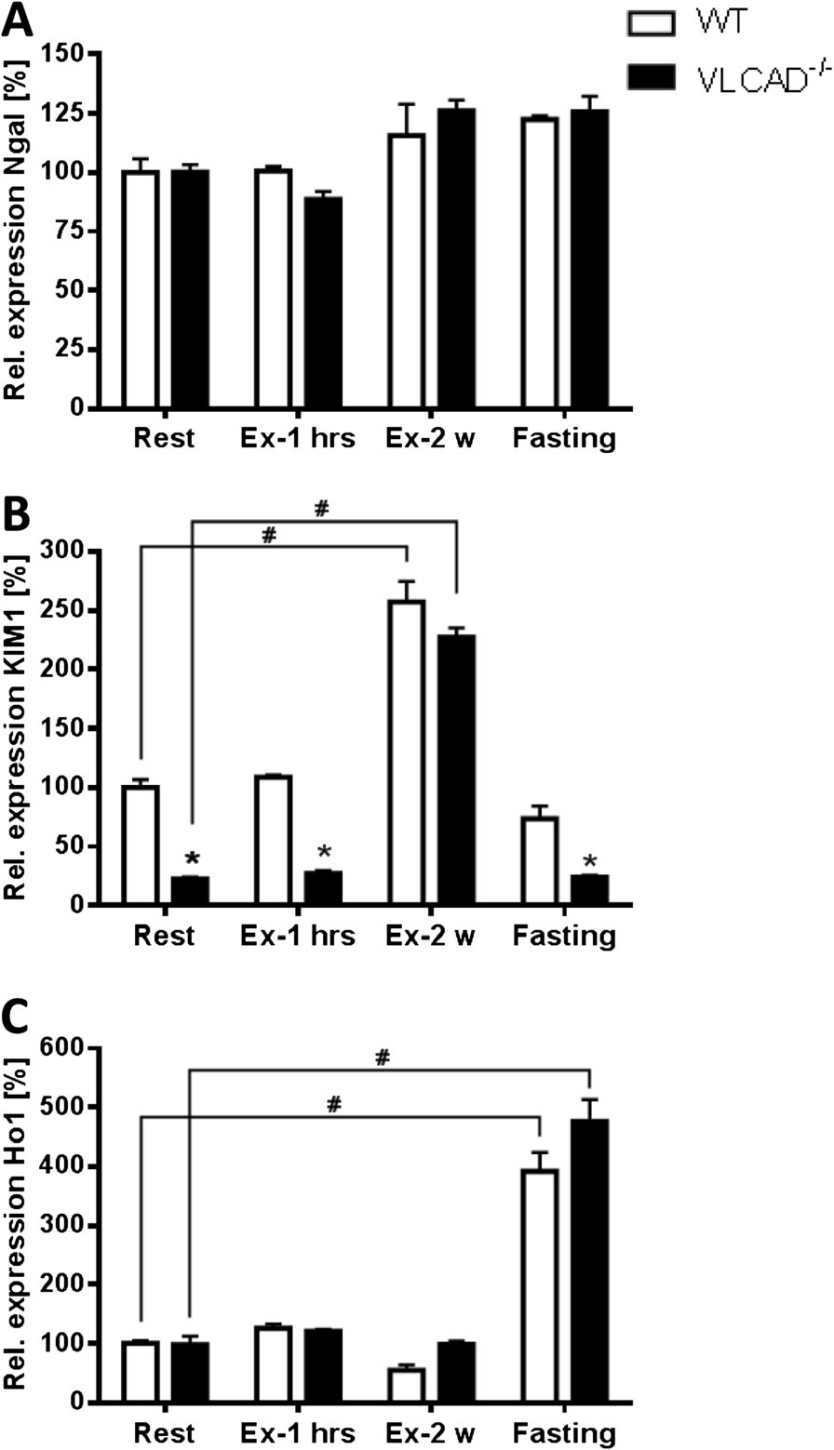
**Expression of genes upregulated during renal failure. (A)** *Ngal*, lipocalin; **(B)** *KIM1*, kidney injury molecule 1; **(C)** *Ho1*, heme oxygenase 1. White and black bars represent WT and VLCAD^−/−^ mice, respectively. Values are represented as mean ± SEM (*n* = 5 to 6). Asterisk indicates significant differences between WT and VLCAD^−/−^ mice within an experimental set. Number sign indicates significant differences between WT or VLCAD^−/−^ mice under different stress conditions as compared to resting mice. Asterisk and number sign denote that values were considered significant if *p* < 0.05 (two-way ANOVA with Bonferroni correction and Student's *t* test).

To appoint the development of oxidative stress due to catabolism as occur in VLCAD^−/−^ mice [11], we measured the specific activity of NADPH:quinone oxidoreductase, GPX, and catalase, which are antioxidant enzymes neutralizing oxidative stress of cytosolic, mitochondrial, and peroxisomal origin, respectively. However, neither the activity of NADPH:quinone oxidoreductase nor of catalase was affected by fasting or exercise, (Figure 3A,B), with the exception of GPX activity, which was significantly reduced in the VLCAD^−/−^ mice as compared to WT after the exercise of about 2 weeks (8.51 ± 0.45 vs. 16.97 ± 3.88 U/mg; Figure 3C). These data are in line with the reduced C16-CoA oxidation rate. Moreover, the expression of genes massively upregulated immediately prior to kidney failure, namely, *Ngal*, *KIM1*, and *Ho1*, was unaffected under all applied stress conditions (Figure 4A,B,C).

### Effect of different stressors on glycogen content and long-chain acylcarnitines in the kidney

**Figure 5:**
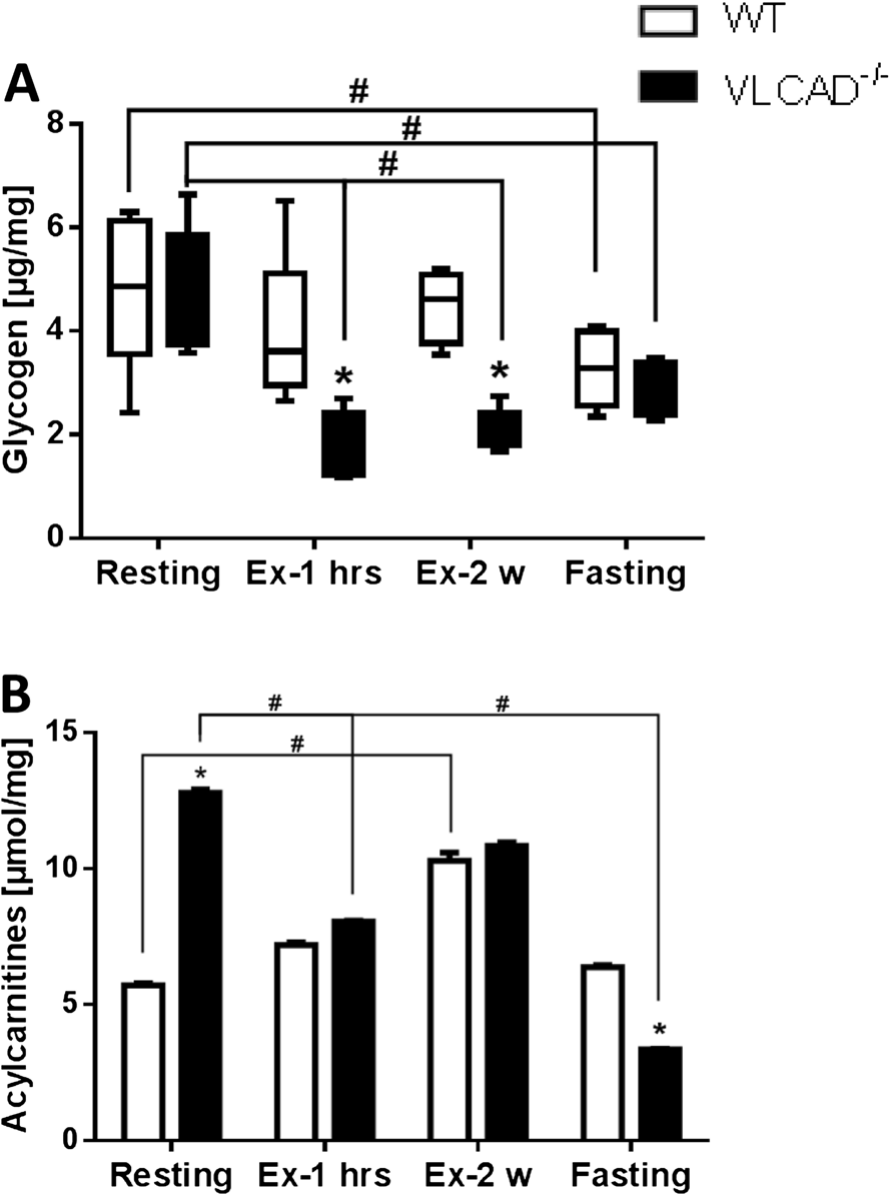
**Renal metabolite content. (A)** Glycogen content and **(B)** long-chain acylcarnitine accumulation in the kidney of WT and VLCAD^−/−^ mice under different stress conditions. White and black bars represent WT and VLCAD^−/−^ mice, respectively. Values are represented as mean ± SEM (*n* = 5 to 6). Asterisk indicates significant differences between WT and VLCAD^−/−^ mice within an experimental set. Number sign indicates significant differences between WT or VLCAD^−/−^ mice under different stress conditions as compared to resting mice. Asterisk and number sign denote that values were considered significant if *p* < 0.05 (two-way ANOVA with Bonferroni correction and Student's *t* test).

In a situation of increased energy demand such as during physical exercise, both glucose and glycogen strongly contribute to the energy supply. We therefore measured the glycogen content in the kidney under resting conditions, after physical exercise, and after 24 h of fasting. Under resting conditions, the glycogen content did not differ between the WT and VLCAD^−/−^ mice. However, after physical exercise, glycogen was more than twofold reduced in the VLCAD^−/−^ mice as shown in Figure 5A. After fasting, the glycogen content was also reduced in both genotypes although significantly only in the mutants.

Acylcarnitines reflect the efficiency of mitochondrial β-oxidation. The analysis of long-chain acylcarnitines in kidney tissue revealed that under resting conditions, the content of these metabolites was more than twofold higher in the VLCAD^−/−^ mice as compared to the littermates (12.8 ± 0.13 vs. 5.7 ± 0.07 μmol/mg; Figure 5B). Of interest was the significant reduction of acylcarnitine content in the VLCAD^−/−^ mice after 24 h of fasting, suggesting that under this condition the kidney must rely on a different energy source more likely glucose.

## Discussion

In this study, we demonstrate that the kidney in the VLCAD^−/−^ mice fully compensates for a defective fatty acid oxidation most likely enhancing glucose oxidation. In contrast to the liver, the main gluconeogenic organ, fasting does not result in an increased renal lipid accumulation and lipogenesis is not induced. In fact, neither signs of oxidative stress nor renal failure are observed during catabolic situations. Importantly, especially long-term exercise promotes a substrate switch so that the kidneys fully rely on glycogen as main source for energy production, while the contribution of fatty acid oxidation is rather minor.

As under catabolic conditions the mice develop hepatic and muscular symptoms [10-12], we hypothesized the same for the kidneys despite the existence of an enzyme with overlapping substrate specificity, the long-chain acyl-CoA dehydrogenase (LCAD) [6,15]. Surprisingly, the C16-CoA oxidation rate was significantly reduced after exercise or fasting, suggesting that the kidney does not rely on fatty acid oxidation during catabolism. Because we expected a renal lipid accumulation as we previously observed in the liver [11], we tested whether lipogenesis was upregulated. Surprisingly, although the VLCAD^−/−^ mice develop a marked steatosis after 24 h of fasting, the kidneys were fully unaffected as shown by the strong downregulation of lipogenic genes. These data are also in contrast to previous reports on the diet-induced obesity mouse model, in which obesity and hepatic steatosis are associated with glomerulosclerosis due to an upregulation of *SREBP-1c* and its target genes [26]. Moreover, in strong contrast to the liver [11] was the finding that catabolic situations *per se* do not represent a trigger able to induce oxidative stress. In fact, the measurement of antioxidant enzyme activities in the three different cellular compartments: mitochondria, peroxisomes, and cytosol, did not reveal renal damage in the VLCAD^−/−^ mice. Consistent with these results was the expression of genes considered reliable markers for renal failure, such as *Ngal*, *KIM1*, and *Ho1*. In fact, the expression of these genes would be highly increased during renal failure even before the corresponding proteins are detectable in serum or urine [27]. The upregulation of *KIM1* and of *Ho1* after prolonged exercise and fasting, respectively, was to ascribe to a genotype-independent physiological response as it occurred at the same degree also in the WT mice.

In the literature, only two cases of myoglobinuric acute renal failure in VLCAD-deficient patients have been reported [28]. In both cases, the diagnosis of acute renal failure was secondary to rhabdomyolysis and myoglobinuria after moderate physical exercise. With the absence of rhabdomyolysis, as shown here, we can demonstrate that the kidneys of the VLCAD^−/−^ mice are not clinically affected.

Kidneys play a distinctive role in glucose homeostasis through glucose filtration, reabsorption, consumption, and gluconeogenesis. In particular, the last process contributes up to 40% to the whole-body glycogen biosynthesis, leaving the kidney a gluconeogenic organ *in vivo* as important as the liver [29,30]. Especially during fasting when glycogen store are depleted and gluconeogenesis becomes the most important process for sustaining the supply of glucose, the kidneys increase their net contribution to glucose release [31]. Although we did not measure glycogen biosynthesis, we observed that during catabolic situations, the glycogen content in VLCAD^−/−^ mice dramatically reduced probably to maintain whole-body glucose homeostasis. These data strongly correlate with the accumulation of long-chain acylcarnitines in many organs which reflect the induced mitochondrial β-oxidation during catabolism but an ineffective oxidation of fatty acids. Very surprisingly, we observed an important correlation between glycogen content and long-chain acylcarnitine accumulation in contrast to other organs [10,11], indicating that during situations of increased energy demand, the kidney mostly likely relies on glucose oxidation. Comparable results have been already described for the skeletal muscle of 1-year-old VLCAD^−/−^ mice under resting conditions [15]. Here, the enhanced glucose oxidation as a compensatory mechanism for a defective FAO machinery was associated with a strongly reduced content of long-chain acylcarnitine.

## Conclusions

Our study shows that under different stress situations, the potential of renal gluconeogenesis can fully compensate the increased energy demand protecting the organ from toxic accumulation of acylcarnitines. The acute renal failure as described in single-case reports appears to be secondary to rhabdomyolysis and myoglobinuria.

## Abbreviations

ESI-MS/MS: electrospray ionization tandem mass spectrometry
FAO: fatty acid oxidation
VLCAD: very long-chain acyl-CoA dehydrogenase
VLCAD^−/−^ mice: very-long-chain acyl-CoA dehydrogenase-deficient mice
WT: wild type
